# Toward the production of block copolymers in microbial cells: achievements and perspectives

**DOI:** 10.1007/s00253-023-12973-8

**Published:** 2024-01-22

**Authors:** Ken’ichiro Matsumoto

**Affiliations:** Division of Applied Chemistry, Faculty of Engineering, Hokkaido University, Kitaku, Sapporo N13W8060-8628 Japan

**Keywords:** Polyhydroxybutyrate, Block copolyester, PHA synthase, PDLA, Biodegradable plastic

## Abstract

**Abstract:**

The microbial production of polyhydroxyalkanoate (PHA) block copolymers has attracted research interests because they can be expected to exhibit excellent physical properties. Although post-polymerization conjugation and/or extension have been used for PHA block copolymer synthesis, the discovery of the first sequence-regulating PHA synthase, PhaC_AR_, enabled the direct synthesis of PHA–PHA type block copolymers in microbial cells. PhaC_AR_ spontaneously synthesizes block copolymers from a mixture of substrates. To date, *Escherichia coli* and *Ralstonia eutropha* have been used as host strains, and therefore, sequence regulation is not a host-specific phenomenon. The monomer sequence greatly influences the physical properties of the polymer. For example, a random copolymer of 3-hydroxybutyrate and 2-hydroxybutyrate deforms plastically, while a block copolymer of approximately the same composition exhibits elastic deformation. The structure of the PHA block copolymer can be expanded by in vitro evolution of the sequence-regulating PHA synthase. An engineered variant of PhaC_AR_ can synthesize poly(d-lactate) as a block copolymer component, which allows for greater flexibility in the molecular design of block copolymers. Therefore, creating sequence-regulating PHA synthases with a further broadened substrate range will expand the variety of properties of PHA materials. This review summarizes and discusses the sequence-regulating PHA synthase, analytical methods for verifying block sequence, properties of block copolymers, and mechanisms of sequence regulation.

**Key points:**

• *Spontaneous monomer sequence regulation generates block copolymers*

• *Poly(D-lactate) segment can be synthesized using a block copolymerization system*

• *Block copolymers exhibit characteristic properties*

**Graphical abstract:**

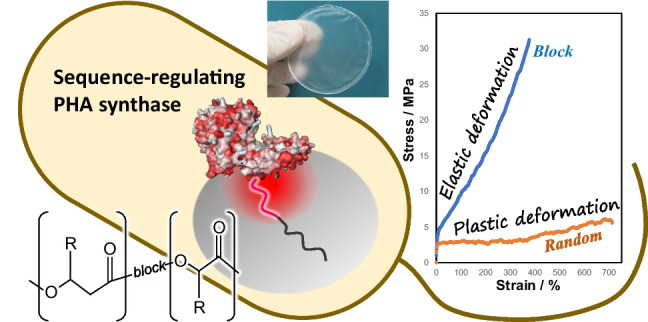

## Introduction

Polyhydroxyalkanoates (PHA) are bacterial storage polyester accumulated as intracellular insoluble inclusions (Zhang et al. 2022). PHAs are synthesized from various hydroxyacyl-coenzyme A (CoA)s via successive ester exchange reactions catalyzed by PHA synthases. PHA synthases are classified into four major classes based on their subunit structure and the range of substrates. The range of substrate of PHA synthase is a key factor in determining the structure of PHA (Neoh et al. 2022).

Research interest in PHAs has increased in recent years due to their biobased and biodegradable thermoplastics applications. The most abundant PHAs are poly(3-hydroxybutyrate) [P(3HB)] and its copolymer with 3-hydroxyvalerate (3HV) (PHBV). Although the brittleness of these polymers has long been recognized as a weakness, recent advances in processing technology have allowed them to be used as a hard material for containers and other applications (Pandey et al. 2022). However, the characterizations of many commercial PHAs are yet to be published. The random copolymer P[3HB-*co*-3-hydroxyhexanoate (3HHx)] (PHBH) is a material whose brittleness is improved by reducing its crystallinity (Tang et al. 2022). PHBH is used to manufacture products such as bags, straws, utensils, and brushes. 4-hydroxybutyrate (4HB)-based polymers are also attracting significant research interest due to their high flexibility and bioabsorbability (Utsunomia et al. 2020). As these studies demonstrate, random copolymerization and composite are the current major strategies to regulate the physical properties of PHAs.

Block copolymers have attracted research attention in polymer chemistry for their ability to form microphase separation by self-assembly. Due to immiscibility and chain connectivity, the phase separation between two or more segments occurs in the tens of nanometers range (Kim et al. 2010). The structure of the microdomains, such as lamellae, cylinders, and spheres, changes depending on the monomer composition and degree of segregation. Block copolymers exert characteristic properties due to microphase separation (Hillmyer and Tolman 2014).

Since the usefulness of block copolymers has been well known in the field of synthetic polymers, it makes sense that interest and attempts to synthesize block copolymers were made in the biosynthesis of PHAs. However, the PHA block copolymer biosynthesis was more complex than initially thought. This review discusses attempts to synthesize block copolymers, the discovery of sequence-regulating PHA synthase, analytical methods for verifying block sequence, properties of block copolymers, and mechanisms of sequence regulation.

## Post-polymerization conjugation and/or extension for block copolymer synthesis

**Fig. 1:**
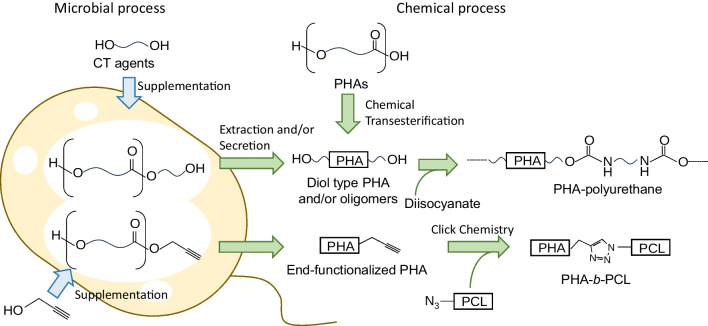
Post-polymerization strategies for block copolymer synthesis. Chain-transfer (CT) agents are attached to the carboxy terminal of PHA. When PEG is used, the obtained polymer is a PHA–PEG block copolymer. End-functionalized PHAs can be assembled into block copolymers using chemical processes

PHA block copolymers are categorized into two major types: block copolymers composed of only PHA segments (PHA–PHA type) and block copolymers of PHA and non-PHA segments. PHA block copolymers containing non-PHA segments were known before PHA–PHA type polymers were reported. Certain alcoholic compounds have been known to serve as chain-transfer (CT) agents in vivo (Fig. 1) (Tomizawa et al. 2013). The CT reaction generates polymers in which the carboxy end is esterified with the CT agents. The use of medium-molecular-weight CT agents, such as polyethylene glycol (PEG), yields PHA–PEG block copolymers, which have a hydroxy group at both ends (diol type) (Shi et al. 1996). Another unique PHA block copolymer synthesized in vivo is the polyphosphate–protein–PHA block copolymer synthesized by using a fusion of PHA synthase and polyphosphate kinase (Hildenbrand et al. 2019).

Diol-type PHAs were also used as components of block copolymers (Fig. 1). The addition of low-molecular-weight CT agents, such as ethylene glycol, to the culture medium generated diol-type PHA (Tomizawa et al. 2010). The use of diethylene glycol in P[lactate (LA)-*co*-3HB)]-synthetic systems generates oligomeric, i.e., low to medium-molecular-weight, diol products. Notably, the oligomers were secreted from bacterial cells into the culture medium, unlike other high-molecular-weight PHAs accumulated as intracellular inclusions (Utsunomia et al. 2017). The diol-type PHAs can be used as a segment to chemically synthesize polyurethane (Hiroe et al. 2021). The diol-type PHA derivatives were also prepared by chemical transesterification using low-molecular-weight diols. The diol-type PHA segments were assembled into a block copolymer using diisocyanate, in which the segments are connected through a urethane group (Mai et al. 2023); they were also used as initiator for ring-opening polymerization (ROP) of ε-caprolactone for synthesizing P(3HB)-*b*-poly(ε-caprolactone) (PCL) (Dai et al. 2009).

The terminal modification technique was used to conjugate polymers to PHA (Fig. 1). For example, the use of 2-propyn-1-ol in vivo as a CT agent produced a polymer with a terminal propargyl group, which was subsequently conjugated with poly(ε-caprolactone) (PCL) by click chemistry (Oyama et al. 2019). The similar block copolymer P(3HB)-*b*-PCL was obtained by stereoselective-chemocatalytic ROP of diolide and lactones (Tang et al. 2021). The same group also synthesized P(3HB)-*b*-P[3-hydroxyheptanoate (3HHp)]-*b*-P(3HB) by ROP using a diol as an initiator (Westlie et al. 2023).

These examples show that many PHA block copolymers are synthesized via post-polymerization conjugation and/or extension, rather than sequential polymerization of multiple segments. Chemical methods can synthesize block copolymers of various segments and sequences. Conversely, a drawback of these methods is that the molecular weight of polymers ≤ 10^4^. This limitation is partly because block copolymerization is achieved by reactions that use a polymer terminal structure to conjugate. Additionally, transesterification may cause segmentation of block copolymers (Lipik and Abadie 2012).

## Attempts to synthesize PHA–PHA type block copolymers in vivo by sequential polymerization

**Fig. 2:**
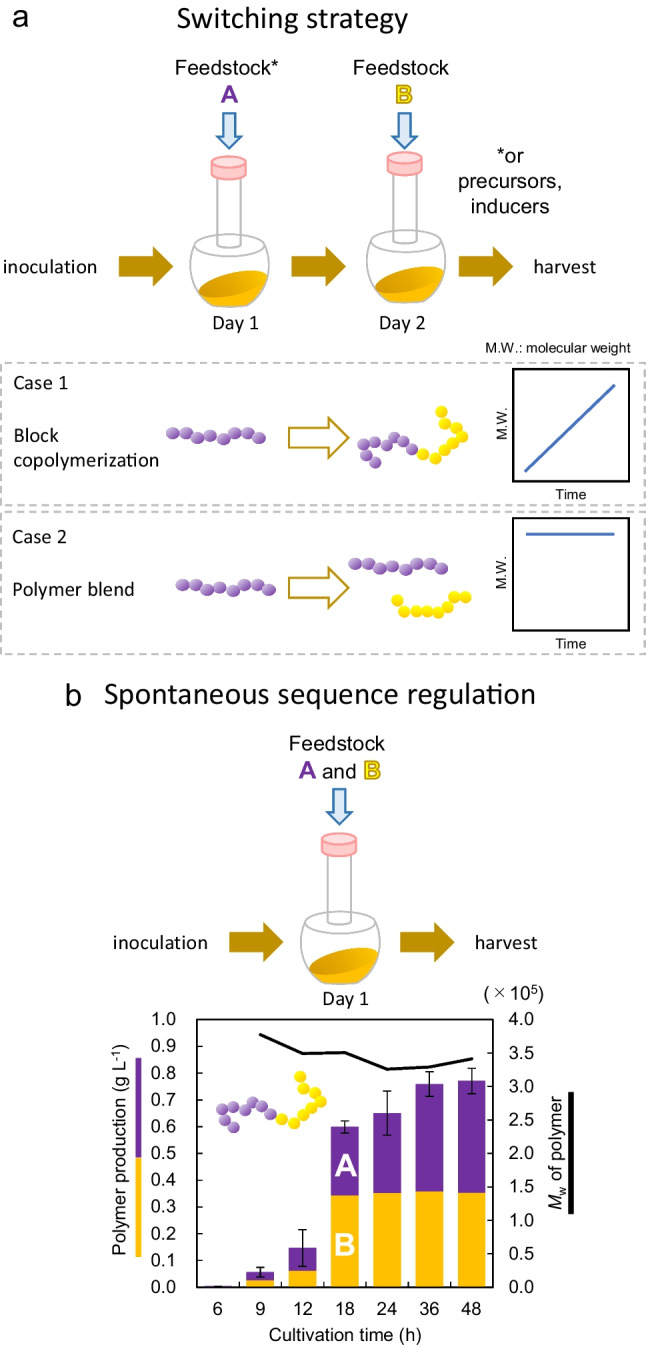
Strategies for block copolymer synthesis. In switching strategies, two feedstocks or inducers (A and B) are added at different times (**a**). If sequential polymerization proceeds, the molecular weight must increase over time (Case 1). However, many PHA biosynthetic systems exhibit nearly constant molecular weight during polymer production, which is likely to synthesize a polymer blend (Cased 2). In contrast, two feedstocks are initially added in the method of spontaneous sequence regulation (**b**). The time profile of the synthesis of the block copolymer P(3HB)-*b*-P(2HB) synthesis using sequence-regulating PHA synthase indicates that monomer units A (3HB) and B (2HB) are simultaneously accumulated. Molecular weight of the polymer is nearly constant during the synthesis. Reproduced from Matsumoto et al. (2018a) with permission

In order to overcome the aforementioned problems, direct synthesis of PHA block copolymers in vivo has been attempted. A simple idea to synthesize PHA block copolymer is sequential polymerization, in which the monomer substrates supplied in the bacterial host are changed during cultivation (switching strategy; Fig. 2a). For example, P(3HB)-*b*-P(3HV-*co*-3HHp) and (3HB)-*b*-P(3HHx) syntheses were attempted by feeding two monomer precursors at different times (Li et al. 2011; Tripathi et al. 2012). As a similar approach, in vivo synthesis of P(3HB)-*b*-P(3HP) was attempted using two induction systems containing isopropyl β-d-thiogalactopyranoside (IPTG)- (for the 3HP pathway) and arabinose-inducible (for the 3HB pathway) promoters (Wang et al. 2013). A problem of the switching strategy is the possibility of random copolymer generation during the changing of monomer supply. To avoid this, Nakaoki et al. harvested the engineered *Ralstonia eutropha* (formally designated as *Cupriavidus necator*) cells once and inoculated them into a fresh medium in an attempt to synthesize PHBV-*b*-P(3HB)-*b*-PHBV (Nakaoki et al. 2019). This approach aims to remove the precursor initially added before adding the next. However, a drawback of these methods is that increasing the number of cultivation steps is challenging. Pederson et al. connected an MS detector to the bioreactor to monitor CO_2_ in the off-gas of the cultivation (Pederson et al. 2006). The decrease in CO_2_ evolution indicates the full consumption of the previously added precursor, and a second precursor was added at this time. Although additional equipment is needed, an advantage of this method is that precursors can be added repeatedly in a short period.

The switching strategy is based on the assumption that the molecular weight of the polymer increases with time (Fig. 2a: Case 1). If sequential polymerization proceeds, the molecular weight of the polymer should increase with each step. However, such an increase in molecular weight at switching has been reported, to the best of my knowledge, only in the attempt of P(3HP)-*b*-P(4HB) synthesis in *E. coli* (Tripathi et al. 2013). It should be noted that the molecular weight of P(3HB) synthesized in *E. coli* did not increase with time, but was nearly constant or rather decreased (Fig. 2a: Case 2) (Kusaka et al. 1997; Tomizawa et al. 2011). This phenomenon occurs because time required to synthesize a single polymer chain is shorter than the cultivation time. Thus, if polymerization stops earlier than the metabolic switch, then polymer blends rather than block copolymers are synthesized. Therefore, we must consider the possibility of polymer blends being synthesized by the switching strategy.

## Sequence-regulating PHA synthase

A breakthrough in achieving PHA block copolymer biosynthesis was the discovery of sequence-regulating PHA synthase (Matsumoto et al. 2018a). The enzyme can spontaneously synthesize block copolymers from *a mixture of substrates* without any manipulation during the polymerization (Fig. 2b). The existence of sequence-regulating PHA synthase was first suggested during an attempt to synthesize LA-based PHA, P(LA-*co*-3HB), using class I PHA synthase (Ochi et al. 2013). Nuclear magnetic resonance (NMR) analysis of the obtained polymer suggested that LA units were *not* randomly incorporated. However, the polymer production and LA fraction were too low to further analyze the details. The results motivated us to explore class I PHA synthases with similar functions. Then, it was found that a chimeric PHA synthase PhaC_AR_ (see below for the details of the enzyme) can synthesize P(3HB-*co*-2HB) with a good yield and that the copolymer obtained was *not* a random copolymer. As mentioned in the next section, the copolymer was proven to be a block copolymer, P(3HB)-*b*-P(2HB).

PhaC_AR_ was originally constructed during the study to create chimeric fusions composed of different PhaCs. Among several junction sites and enzyme combinations tested, only PhaC_AR_ with 26% N-terminal PhaC_Ac_ from *Aeromonas caviae* and 74% C-terminal PhaC_Re_ from *R. eutropha* exhibited sufficient enzymatic activity (Matsumoto et al. 2009). Nowadays, the prediction of the 3D structure using Alphafold2 accounts for the result. The junction site of PhaC_AR_ locates an interdomain region of the N-terminal and C-terminal domains (Phan et al. 2022). The chimerization generates the sequence-regulating capacity of PhaC_AR_, indicating the importance of N-terminal domain for the function of the enzyme. In fact, the important role of N-terminal domain has been demonstrated by the experimental results showing that deletion of N-terminal few amino acid residues altered the substrate specificity of PhaCs (Lim et al. 2021; Ye et al. 2008). However, the structure-function relationship of the N-terminal domain remains to be elucidated.

The spontaneous sequence regulation was first found on an engineered *Escherichia coli* platform. Recently, the block copolymer P(3HB-*ran*-3HV)-*b*-P(2HB) was successfully synthesized in engineered *R. eutropha* (Ishihara et al. 2023). Therefore, sequence regulation is not a specific phenomenon in *E. coli* and is likely applicable to various platforms.

## Verification of block structure

As previously discussed, a random copolymer and/or polymer blend could be synthesized under the culture conditions, attempting to synthesize block copolymers. Therefore, the distinction between random copolymer, block copolymer, and polymer blends is an important issue.

### NMR analysis of block copolymers

**Fig. 3:**
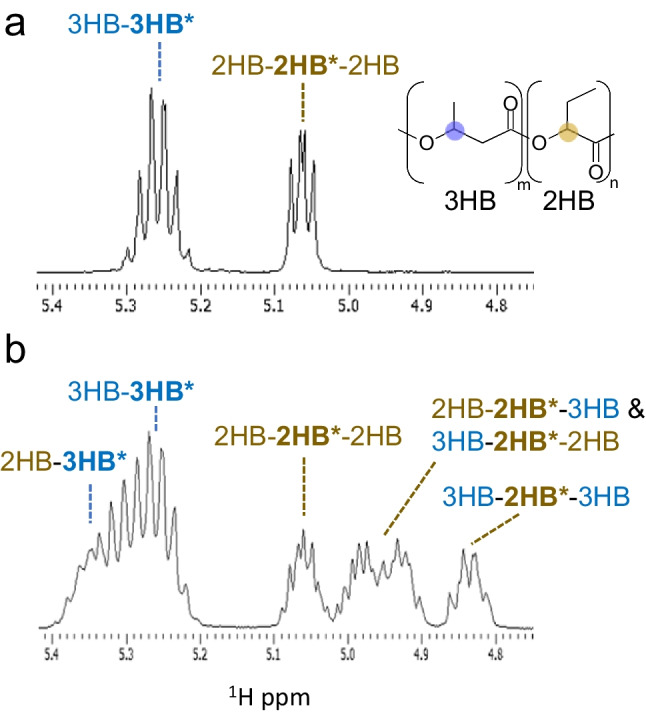
^1^H NMR of the block (**a**) and random (**b**) copolymer of P(3HB-*co*-2HB). The resonances of the methyne proton are magnified. Random copolymers can be clearly distinguished from block copolymers because the signals are observed at different chemical shifts due to the influence of adjacent units. Reproduced from Matsumoto et al. (2018a) with permission

NMR is a common method to evaluate the monomer sequence of polymers. The chemical shift is slightly altered depending on the presence of neighboring units. Since this effect is stronger at closer distances, the signal of the atoms at the edge of the unit, typically carbon from the carbonyl group, is usually used for the sequence analysis of P(3HA)s. Based on this principle, the dyad sequence frequency was determined. In the case of 2-hydroxyalkanoate (2HA) units, the triad sequence can be determined due to the chemical shifts to be affected by both neighboring units (Fig. 3). A parameter D, defined as follows, is used to evaluate the randomness of the monomer sequence where F_AB_ represents the signal intensity of an A unit neighboring a B unit (Kamiya et al. 1989; Bartels et al. 2020).

$$D=\frac{{F}_{AA}{F}_{BB}}{{F}_{AB}{F}_{BA}}$$

Random copolymers would have a *D* value of approximately one independent of their monomer composition (A:B). The *D* value of the block copolymer should be > 1, or is undefined when *F*_AB_ or *F*_BA_ is not detected. The *D* value clearly distinguishes random copolymers from block copolymers. However, it should be noted that there is no significant difference between the *D* values of block copolymers and polymer blends. Therefore, a high (or undefined) *D* value is a necessary, but not sufficient, condition for the existence of a block copolymer. The distinction between the block copolymer and polymer blend is thus important for verifying the block sequence of the polymers.

### SEC and DOSY NMR

**Fig. 4:**
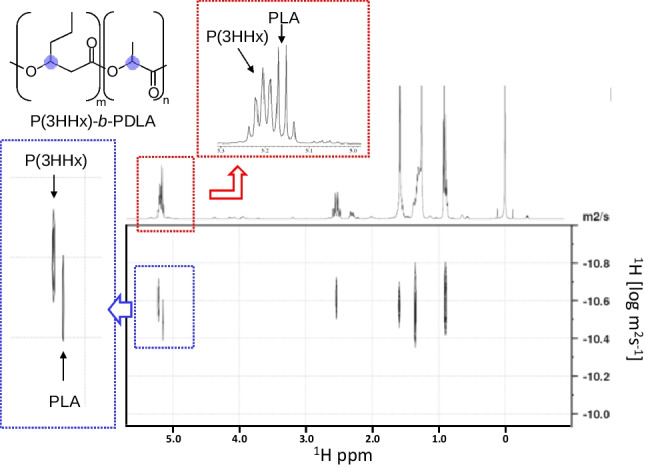
DOSY NMR analysis of PHA block copolymer, P(3HHx)-*b*-PLA. The range of diffusion coefficient of 3HHx and LA units overlapped, indicating that some of these segments can be the same molecule

The polymer’s molecular weight distribution determined by size exclusion chromatography (SEC) is used to distinguish the block copolymer and polymer blend. If bimodal peaks are observed, the polymer is likely a polymer blend. In contrast, the observation of unimodal peaks is a necessary but not sufficient condition for a sample to be a block copolymer. Relatedly, diffusion-ordered spectroscopy (DOSY) NMR analysis is also a useful method (Fig. 4) (Phan et al. 2023). The observation of resonances of each segment at different diffusion coefficients indicates that they are not the same molecule. Otherwise, the sample could be a block copolymer, but this is a necessary condition. DOSY NMR is particularly useful when one segment is a minor fraction and is hardly detected by SEC.

### Solvent fractionation

**Fig. 5:**
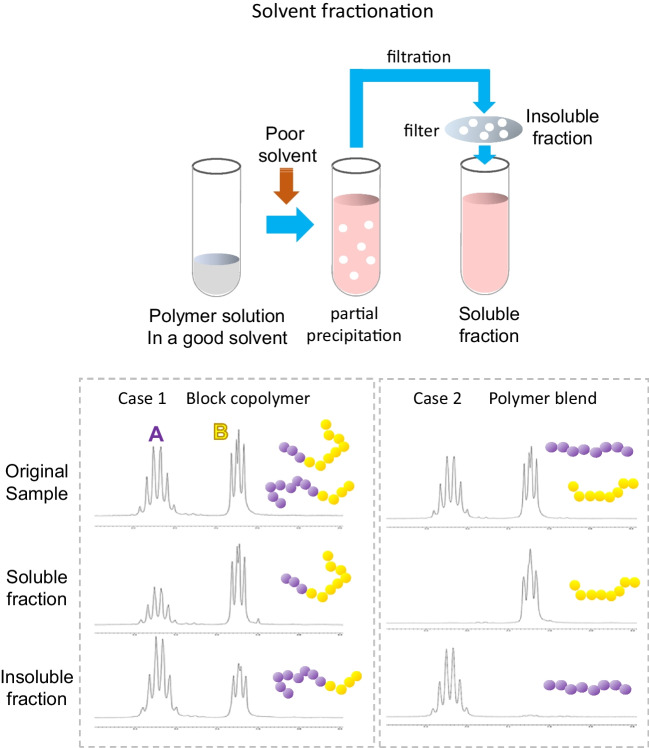
Solvent fractionation of PHA block copolymers and polymer blend. The polymer sample is initially dissolved in a good solvent (both segments are soluble). Subsequently, a poor solvent, in which only one segment is dissolvable, was added to partly precipitate the sample. Then, soluble, and insoluble fractions are separated by filtration. The data is ^1^H NMR of P(3HB)-*b*-P(2HB) and the corresponding blend. These samples are initially dissolved in chloroform (good solvent). In the case of block copolymer, both segments are detected even in poor solvent (tetrahydrofuran) due to the covalent linkage between both segments (Case 1). The polymer blend is completely separated with the same treatment (Case 2)

In the case of sequential polymerization by the chemical methods, block copolymerization can be confirmed by measuring the molecular weight at each step of segment synthesis. However, in the case of in vivo PHA synthesis, this method is not applicable because analysis during polymerization is not possible. Alternatively, solvent fractionation can verify the existence of covalent linkage(s) between segments (Fig. 5). Solvent fractionation is a technique that separates possibly mixed PHAs based on differences in the solubility of their respective parts (Mizuno et al. 2017). In order to apply this method to block copolymerization verification, it is necessary to identify a solvent in which the solubility of each segment differs considerably. Homopolymer blend extractions in such a solvent will solubilize only one of the polymers and the blends will be completely separated. In contrast, block copolymers are not separated by the same treatment. The presence of covalent bonds between the two segments can be confirmed by partially dissolving a segment in an insoluble solvent (Matsumoto et al. 2018a; Phan et al. 2023, 2022; Tomita et al. 2022).

### Microphase separation

**Fig. 6:**
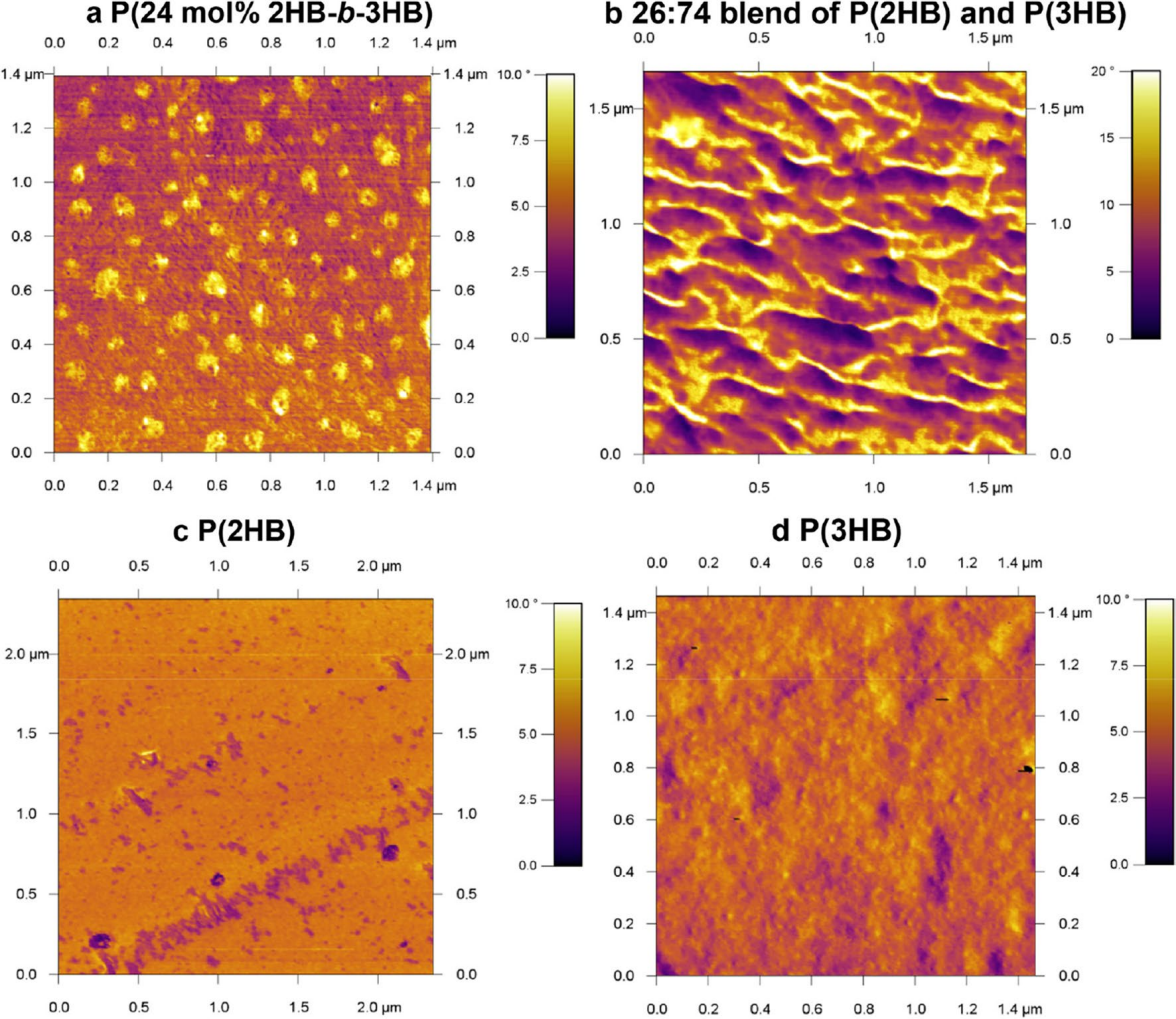
Phase retraced images of the spin-coated films of polymers composed of 2HB and/or 3HB using AFM. Reprinted from Matsumoto et al. (2018a) with permission

The observation of microphase separation is useful for verification of the block sequence, because microphase separation is a characteristic structure of block copolymers, the principle by which these copolymers exhibit their characteristic properties. Atomic force microscopy (AFM) is suitable for observing block copolymers composed of segments with different elastic moduli because it can measure the surface elastic modulus in phase mode. The AFM observation of a 3HB-rich P(3HB)-*b*-P(2HB) spin-coated film demonstrated a sea-island structure with island sizes of approximately 20 nm (Fig. 6a). The structure was not seen in a blend (Fig. 6b) and homopolymers (Fig. 6c and d).

In order to form microphase separation, the segments of the block copolymer must be immiscible each other. The requirement of immiscibility is not easily achieved for PHA–PHA type block copolymers because both segments are aliphatic polyesters with similar structures. Therefore, PhaC_AR_ that can synthesize immiscible P(3HA) and P(2HA) segments is useful for molecular design of block copolymers.

### Thermal properties

The monomer sequence is an important determinant of the physical properties of polymers. Conversely, the phase separation and monomer sequence can be verified on the physical properties. It has been established that the glass transition temperature (*T*_g_) of random copolymers is between the *T*_g_ of the homopolymers of the respective components (Conti et al. 2006). In contrast, block copolymers composed of two incompatible segments exhibit two *T*_g_ values for each polymer. Because incompatible polymer blends also exhibit two *T*_g_ values, they are an indicator of incompatibility but not evidence of block copolymerization.

Similarly, block copolymers comprised of two crystalline segments typically have two melting temperatures (*T*_m_). If two different endothermic peaks are observed by differential scanning calorimetry (DSC), there are at least two crystal regions in the polymer, i.e., phase separation probably occurs. However, the *T*_m_ values of the two components may be close, or altered by block copolymerization so that the *T*_m_ peaks are not clearly observed. In such cases, X-ray diffraction is useful to identify the crystal structures (Phan et al. 2023).

## Influence of block versus random monomer sequence on mechanical properties

**Fig. 7:**
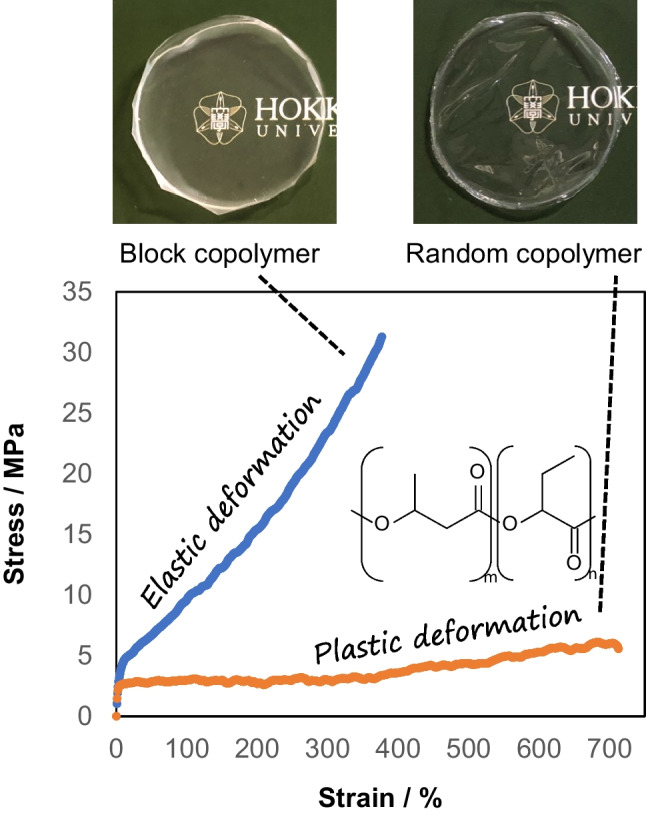
Different mechanical properties of block and random copolymers of P(3HB-*co*-2HB). The block and the random polymer contained 88 and 92 mol% 2HB, respectively. The block copolymer data was recorded at 32 °C; thus, no yield point was observed. Reprinted from Kageyama et al. (2021) with modification under the Creative Commons License

The monomer sequence is one of the major determinants of the mechanical properties of the polymer. The exertion of diverse properties by using the same monomer components is an advantage of sequence regulation. In particular, chemically synthesized ABA-type block copolymers consisting of hard (A) and soft (B) segments are known to be tough and/or elastic (Mulchandani et al. 2021; Maji and Naskar 2022). In the P(3HB-*co*-2HB) example, the random copolymer exhibited plastic deformation, while the block copolymer of similar composition exhibited elastic deformation (Fig. 7) (Kageyama et al. 2021). However, it should be noted that the number of segments in the copolymer synthesized using PhaC_AR_ has not been analyzed. In vitro analysis of PhaC_AR_ suggests that the enzyme is unlikely to synthesize ABA-type block copolymers (see Mechanism analysis of block copolymerization). Therefore, further studies are needed to elucidate the elasticity mechanism of PHA block copolymer.

In combining soft and hard segments, polymerization must be performed by a single enzyme. If two enzymes with different substrate specificities are used, each polymer will be synthesized separately, and block copolymers will not be obtained. Therefore, PHA synthases with a broad substrate range are needed for the synthesis of useful block copolymers. PhaC_AR_ recognizes an extremely broad range of substrates including SCL, MCL, and long-main-chain monomer substrates (see the “Mechanism analysis of block copolymerization” section), which is an advantage for synthesizing block copolymer (Satoh et al. 2022).

Block sequence influences the crystallization rate of polymers. The crystallization rate of P(3HB) is slower than that of many commodity plastics (Eesaee et al. 2022), and block copolymerization further slows crystallization. McChalicher et al. (2007) reported that block copolymerization of 3HB and 3HV decelerated crystallization compared to random copolymers, and as such, the block copolymers retained their extensibility for a longer time than random copolymers (McChalicher and Srienc 2007). A similar phenomenon was observed for P(3HB)-*b*-P(2HB). No P(2HB) crystal was detected in P(3HB)-*b*-46 mol% P(2HB) by DSC measurement of the polymer (Kageyama et al. 2021).

## Directed evolution of sequence-regulating PHA synthase

As mentioned above, sequence-regulating PHA synthases are desirable to possess a broad substrate range capable of synthesizing structurally diverse segments with contrasting physical properties. However, the sequence-regulating capacity has been found only in class I PHA synthases, which have a relatively narrow substrate range toward SCL monomers. These findings have inspired us to evolve the sequence-regulating PHA synthase PhaC_AR_ to increase its activity toward an MCL substrate, 3HHx-CoA (Phan et al. 2022). Therefore, particularly useful mutations N149D and F314H were found through random mutagenesis, high-throughput screening, and subsequent saturation mutagenesis. The mutation N149D is located in the PhaC_Ac_ region of PhaC_AR_, which is the same position to the well-known beneficial mutation N149S in PhaC_Ac_ (Kichise et al. 2002; Kawashima et al. 2015). Notably, the F314H mutant can synthesize P(3HHx) homopolymer. This finding enabled the synthesis of a new type MCL–SCL block copolymer, P(3HHx)-*b*-P(2HB) (Phan et al. 2022).

In this study of enzyme evolution, it was surprising that *all* of saturation mutants (F314X) increased 3HHx fraction in P(3HB-*co*-3HHx) compared to the original PhaC_AR_ with F314, and 16 mutations increased P(3HHx) synthesis (Phan et al. 2022). This fact suggests that the original Phe residue (F314) was selected in natural evolution for the strict specificity toward 3HB-CoA. Thus, the attempts to expand the substrate range of PHA synthase might be a reverse evolution toward the ancestor enzyme with less precise substrate recognition.

## Biosynthesis of PDLA-containing PHA block copolymers

Polylactate (PLA) is the most widely used biobased polymer. PLAs are rigid, biocompatible, and compostable materials typically produced through microbial lactic acid production and chemical polymerization. PLA has several advantageous features over natural PHAs, such as superior transparency, less brittleness than P(3HB), and high lactic acid yield from sugar (Swetha et al. 2023; Ranakoti et al. 2022). Furthermore, poly(d-LA) (PDLA) is an attractive block copolymer component due to the stereo-complex formation with PLLA (Stefaniak and Masek 2021). PLAs are normally obtained by ROP of lactides. The strengths of the PHA biosynthetic system are, for example, one-pot fermentation production using various feedstock, strict isotactic polymerization (toward D isomer), and high molecular weight of polymers. Because of these benefits and structural similarities between PLA and PHA, PLA biosynthesis using a PHA production system has attracted research interest (Valentin and Steinbüchel 1994). However, PHA synthases capable of incorporating LA units have not been found for a long time.

The first LA-CoA-polymerizing enzyme, PhaC1_Ps_STQK, was reported in 2008, which is an engineered class II enzyme from *Pseudomonas* sp. 61-3 with S325T/Q481K pairwise mutations (Taguchi et al. 2008). The incorporation of LA units is d-LA specific, as well as other natural PHA monomers (Yamada et al. 2009). The discovery enabled the synthesis of d-LA-based random copolymers from various nonrelated carbon sources, such as xylose (Nduko et al. 2013), xylan (Salamanca-Cardona et al. 2016), and corn stover hydrolysate (Wu et al. 2021).

An important feature of PhaC1_Ps_STQK and its homologous enzymes is that they synthesize only a random copolymer but PLA homopolymer and LA-rich copolymers are synthesized only with very low yield and molecular weight. For example, P(96 mol% LA-*co*-3HB-*co*-3HV) was synthesized in *E. coli* with a 0.4 wt% content and weight-average molecular weight (*M*_w_) of 1 × 10^4^ (Shozui et al. 2011). The engineered *Corynebacterium glutamicum* expressing PhaC1_Ps_STQK and propionyl-CoA transferase produced P(99 mol% LA-*co*-3HB) with 1 wt% content and *M*_w_ of 6 × 10^3^ (Song et al. 2012). Similar results were obtained using *E. coli* (4 wt% PLA) (Jung and Lee 2011) and yeasts [3 wt% PLA, *M*_w_ of 5 × 10^4^ (Lajus et al. 2020) and 4 wt% PLA, *M*_w_ of 5 × 10^3^ (Ylinen et al. 2022)] expressing engineered PHA synthases homologous to PhaC1_Ps_STQK. Using an in vitro polymer synthetic system, a PhaC1_Ps_STQK homologous mutant from *Pseudomonas* sp. SG4502 did not synthesize PDLA (Tajima et al. 2012). Class I PHA synthase from *Chromobacterium* sp. USM2 also synthesized low molecular weight PLA with low production (2 wt% and *M*_w_ of 2 × 10^4^) (Shi et al. 2022). Given the common polymer content and molecular weight (for example, > 30 wt% and *M*_w_ of 10^5^–10^6^, in typical engineered *E. coli*) of PHA, PLA biosynthesis is strictly limited.

For the first time, the biosynthesis of high-molecular-weight PDLA was achieved by utilizing a block copolymerization system (Phan et al. 2023). PhaC_AR_ variants F314H (FH) and N149D/F314H (NDFH) were found to efficiently incorporate LA units into the polymer chain under copolymer synthesis conditions. These enzymes synthesized P(3HB)-*b*-PDLA and P(3HHx)-*b*-PDLA block copolymers. However, none of the original and variant PhaC_AR_ synthesized PDLA homopolymers.

The molecular weight of P(3HHx)-*b*-PDLA is 10^5^ in order of magnitude. The number of segments in the PHA block copolymer, namely the diblock, the triblock, or more, remains undetermined. Therefore, it is impossible to calculate the PDLA segment’s molecular weight. However, the PHA block copolymers are unlikely to contain many segments based on their crystallization behavior and kinetic analysis of block copolymerization (see Mechanism analysis of block copolymerization). Assuming the above, the molecular weight of PDLA segment can be 10^5^ (or close to 10^5^) in order of magnitude, which is larger than the upper limit demonstrated previously.

### Mechanism of the PLA biosynthesis

**Fig. 8:**
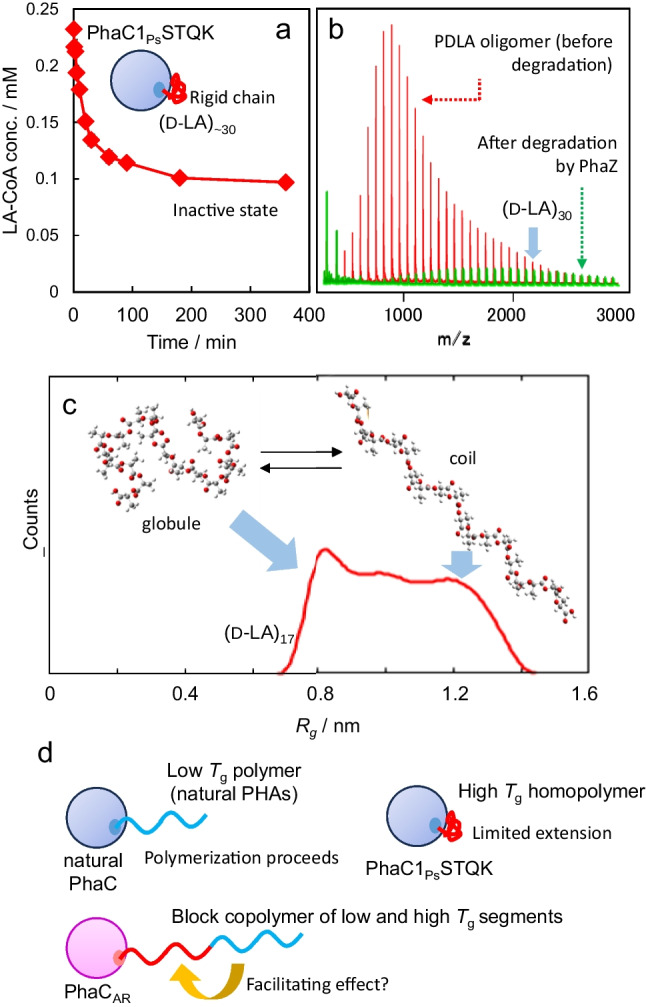
Polymerization and depolymerization of PDLA oligomers. In vitro analysis of PLA synthesis by PhaC1_Ps_STQK indicated that d-LA-CoA consumption was stopped while the substrate remained (**a**). The rigid PLA product likely blocks the substrate binding site of the enzyme and inactivates the enzyme. The molecular weight of the product (PLA) is approximately 2000 (DP ~ 30). A related phenomenon was observed in the degradation of PDLA by PhaZ (**b**). PhaZ hardly degrades the PDLA oligomer larger than an approximate DP of 30. The agreement of the upper limits of DP of polymerization and depolymerization of PDLA indicates that there is a common mechanism behind the phenomena. Molecular dynamics simulation of the PDLA oligomer indicates that the conformation of PDLA oligomers changes depending on their DP (**c**). PDLAs of DP 30 are mostly globule-forming. Therefore, the inability of PDLA biosynthesis is likely due to the physicochemical properties of PDLA oligomers. Proposed model of PDLA segment synthesis by PHA synthase (d). The *T*_g_ values of natural PHAs are lower than cultivation temperature. Thus, polymerization proceeds smoothly. In contrast, the extension of polymer chains of artificial PHAs, such as PDLA, with high *T*_g_ is limited, presumably due to the low mobility of the polymerized product. PDLA-containing block copolymer synthesis is possible, maybe because the low *T*_g_ segment facilitates the extension of the high *T*_g_ segment. Reprinted from Matsumoto et al. (2018b) and Hori et al. (2020) with permission

These results raise the question as to why the PDLA segment synthesis is possible, while the homopolymer is hardly obtained. The limitation has often been attributed to the low activity of the enzymes toward LA-CoA or weak LA monomer supply. However, the in vitro kinetic analysis of PhaC1_Ps_STQK has revealed that the assumption is not necessarily correct (Matsumoto et al. 2018b). In fact, the initial LA-CoA reaction rate is comparable to that of 3HB-CoA. However, observations showed that the LA-CoA consumption stops, although the substrate is still present (Fig. 8a). The enzyme at this stage is in an inactive state and cannot react even with the preferable monomer 3HB-CoA. The reaction product is PLA with a molecular weight of 2 × 10^3^ [degree of polymerization (DP) is approximately 30], indicating that when the molecular weight reaches this range, the polymerization becomes very slow. Studies on the enzymatic degradation of d-LA containing PHAs revealed that PhaZ does not degrade PDLA oligomers greater than approximately DP 30 (Fig. 8b) (Sun et al. 2015). The coincidence of upper molecular size limits of PDLA oligomers for synthesis and degradation indicates that these phenomena are due to a common mechanism. The results of the molecular dynamics simulation indicate that the conformation of the PLA oligomer changes depending on the degree of polymerization, and PLA chains longer than DP 30 take an aggregated conformation (Fig. 8c) (Hori et al. 2020). Indeed, *T*_g_ of PLA is 60 °C; thus, PLA chains are stiff at cultivation temperatures (typically 30 °C).

The relationship between *T*_g_ and the cultivation temperature was verified for P(2HB) synthesis, *T*_g_ of which is 30 °C. Production and molecular weight of P(2HB) considerably increased at cultivation temperatures higher than 32 °C (Matsumoto and Kageyama 2019). Therefore, the inefficiency of PLA synthesis is not due to low enzyme activity but rather to the physicochemical properties of the polymerized product. Because only PDLA synthesis as a block copolymer segment is possible, P(3HHx) segment should play an important role. The presence of the segment with low *T*_g_ [− 28 °C for P(3HHx)] may facilitate the extension of the PDLA chain (Fig. 8d). More analysis is needed to clarify the mechanism of synthesis and the role of P(3HHx) segment.

## Mechanism analysis of block copolymerization

The discovery of spontaneous block copolymerization raised questions about the mechanism, such as under what synthetic conditions does sequence regulation occur? Two substrates are believed to be present in the cells. If so, why is one substrate not incorporated during the synthesis of the other segment? What is the factor that causes the segment to switch?

### 2HA monomers are essential for block copolymer synthesis

**Fig. 9:**
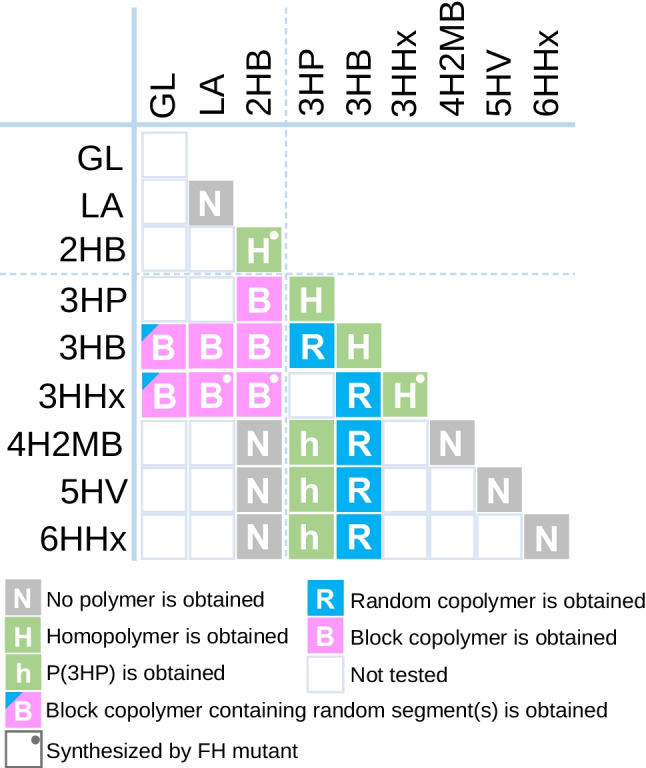
Regularity of the monomer sequence synthesized using the PhaC_AR_ and/or F314H mutant by a combination of monomers. The table indicates the combinations of monomer precursors (Arai et al. 2020; Phan et al. 2023, 2022; Satoh et al. 2022). The combination of GL and 3HHx indicates ternary copolymer-producing conditions containing 3HB (Tomita et al. 2022). 4H2MB, 4-hydroxy-2-methylbutyrate

PhaC_AR_ synthesizes block copolymers but also can produce random copolymers. This phenomenon raised the question of factor(s) in monomer structures that determine their sequence. In order to answer this question, various combinations of monomers were tested, and the monomer sequence of the obtained polymers was analyzed. PhaC_AR_ recognizes hydroxyacyl-CoA with a wide range of main-chain-length from C_2_ to C_6_. Long-main-chain substrates, such as 5-hydroxyvalerate (5HV) and 6-hydroxyhexanote (6HHx), are incorporated into the polymer as copolymers with 3HB units, and their homopolymers were not synthesized. The obtained copolymers possess a random sequence. Among the conditions tested, the presence of 2HA monomers was found to be essential for the block copolymer synthesis (Fig. 9) (Satoh et al. 2022; Phan et al. 2022). Using FH mutant, P(3HHx) homopolymer, and its block copolymers, P(3HHx)-*b*-P(2HB) and P(3HHx)-*b*-PDLA, were synthesized. When three monomers are combined, a block sequence is generated between 2HA-containing and non-2HA-containing segments. For example, the combination of 3HB, 3HV, and 2HB yields P(3HB-*ran*-3HV)-*b*-P(2HB) (Ishihara et al. 2023), and combining glycolate (GL) and 3HB yields P(3HB)-*b*-P(GL-*ran*-3HB) (Arai et al. 2020). These results indicate that 2HA-CoA is important in determining the monomer sequence. The analysis of the mechanism of block copolymer synthesis will be discussed in the next section. However, the reason for the special effect of 2HA-CoA remains to be elucidated.

### In vitro analyses

**Fig. 10:**
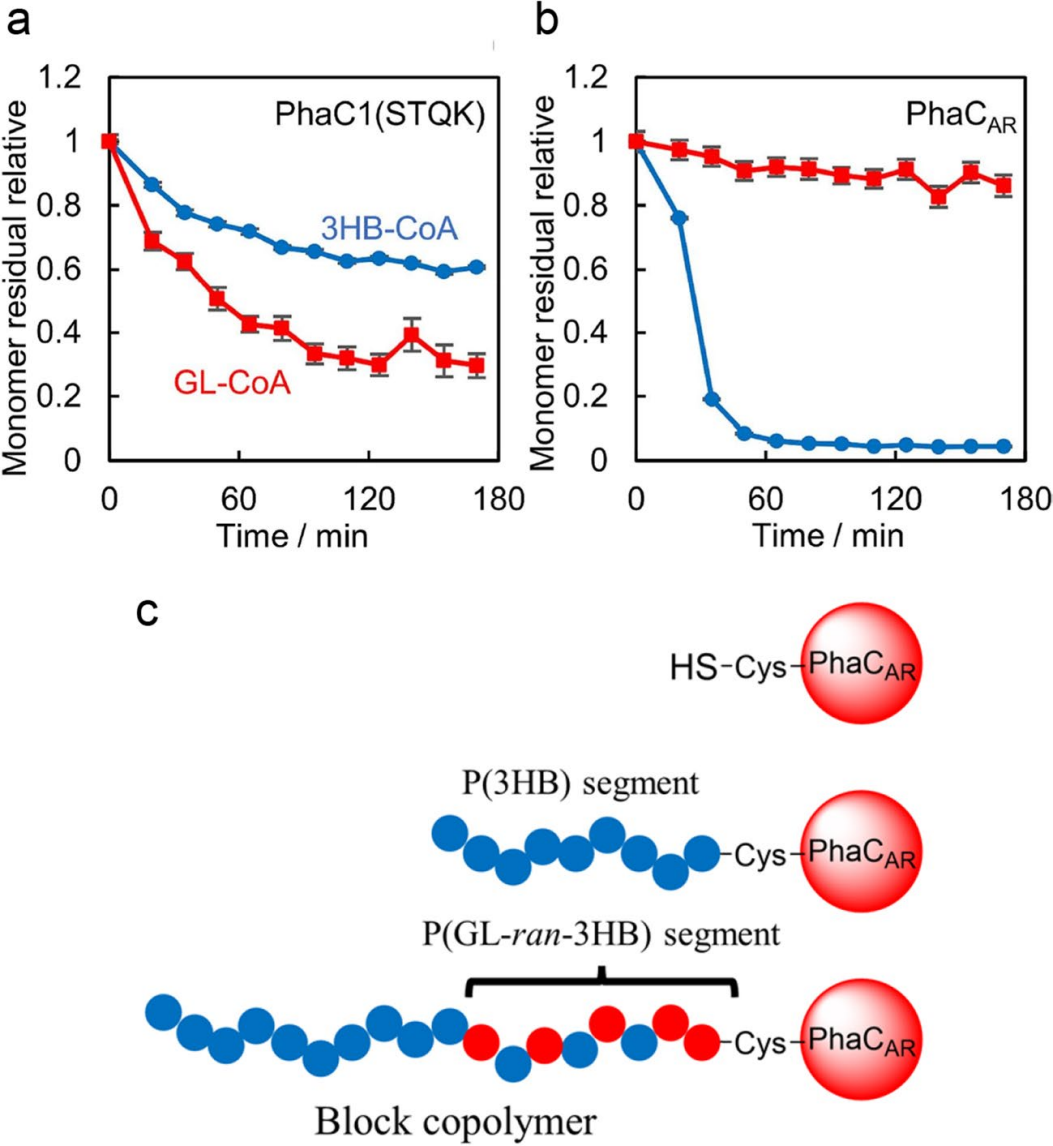
Real-time NMR analysis of the copolymerization of GL-CoA and 3HB-CoA by PhaC1_Ps_STQK (**a**) and PhaC_AR_ (**b**) and proposed scheme of block copolymerization (**c**). Reprinted from Yanagawa et al. (2023) with permission

The block sequence tends to be thought to result from the difference in reaction rates of the two substrates. However, the simple model does not fully explain the mechanism of block copolymerization of PHAs. For example, PhaC_AR_ activity toward 3HB-CoA is higher than that toward 3HHx-CoA (Phan et al. 2022), but the obtained product is a random copolymer P(3HB-*ran*-3HHx). Therefore, in vitro analysis of PhaC_AR_ was conducted to elucidate the mechanism of block copolymerization. There was a technical barrier to measuring the copolymerization rate. Normally, the amount of CoA released is used as an indicator of polymerization progress (Tan et al. 2020). However, the principle is not applicable to copolymerization. Therefore, the consumption of multiple substrates was determined by periodic measurements of the reaction mixture using liquid chromatography mass spectroscopy (LC-MS) (Matsumoto et al. 2018a). The real-time NMR technique is also applicable to measure copolymerization. By using 800-MHz high-sensitivity NMR and ^13^C-labeled monomer substrates, acquisition time can be as short as 15 min, which allows monitoring of the progress of the reaction in an NMR machine (Fig. 10a, b) (Yanagawa et al. 2023). An advantage of this method is that unidentified soluble product(s) can be detected compared to the LC-MS method, in which the m/z of the target molecule must be known to quantify the product(s). Note that the polymerized product is insoluble in water, which makes it not detectable by liquid NMR.

These experiments revealed that (i) the original PhaC_AR_ initially reacts very little with 2HA-CoA (2HB-CoA and GL-CoA); (ii) under the two substrate conditions, only 3HB-CoA is consumed at the initial stage of the reaction; (iii) after the consumption of 3HB-CoA, 2HA-CoA starts to be consumed. The result indicates that PhaC_AR_ acquires activity toward 2HA-CoA during the reaction (Fig. 10c). In contrast, PhaC1_Ps_STQK exhibited activity toward 2HA-CoA at the initial reaction stage (Fig. 10a). The initial very low activity of PhaC_AR_ toward 2HA-CoA is key for block copolymerization. Additionally, these results demonstrated that the sequence regulation is, at least partly, attributed to the function of PHA synthase. However, the contribution(s) of other enzymes is not ruled out. For example, *E. coli* possesses LA-CoA-degrading enzyme(s), which effects on the LA-containing polymer synthesis (Matsumoto et al. 2018b). Further studies are needed to understand the role of these enzymes in sequence regulation.

## Perspective

PHA block copolymer biosynthesis is a promising technology that can expand the properties of PHA, that is, its range of applications. Studies on P(3HB)-*b*-P(2HB) have shown the influence of block sequence on physical properties. Further exploration of block copolymer structures and the development of materials are needed. Combining soft and hard segments is a common and effective molecular design of block copolymers. To meet this goal, PDLA is a potent structure as a hard segment.

The construction of de novo synthetic routes is required in terms of the development of biobased plastic production. Currently, many block copolymer synthetic systems utilize precursors and/or related carbon sources supplemented in the medium. The polymer production using this method is stable and reproducible; the monomer composition can be facilely controlled. Therefore, the method is useful for exploring novel polymer structures. On the other hand, a disadvantage is toxicity of organic acid. Thus, the precursor concentration in the medium must be controlled below the concentrations at which microbial growth is inhibited. To overcome the problem and to utilize biomass, the construction of monomer-supplying routes from nonrelated carbon sources, such as sugars, are needed. Recently, the production of P(3HB-*ran*-3HV)-*b*-P(2HB) was successfully synthesized in engineered *R. eutropha* (Ishihara et al. 2023). This is important progress toward an industrial process for block copolymer production. The development of the molecular design of polymers, biosynthetic routes, and microbial strains will make it possible to add PHA block copolymers to the list of practical PHAs.

One of advantages of the block copolymer biosynthesis system over chemical methods, such as ROP, is high molecular weight of the polymers. In many cases, the molecular weight of chemically synthesized polyesters by ROP is limited ≤ 10^4^ in order of magnitude (McMichael et al. 2023; Saar and Lienkamp 2020). Furthermore, the strict chiral specificity of PHA synthases enables the synthesis of isotactic polymers. On the other hand, a drawback of the biosynthesis is the limited substrate range of PHA synthase. Another limitation is the lack of control over the order of segments. Therefore, expanding the function of sequence-regulating PHA synthase will be an important research target.

The effect of block sequence on biodegradability is also of interest. As block copolymers synthesized using PhaC_AR_ and its variants contain non-natural units, their biodegradability in various environments, including marine environments may differ from that of natural PHAs. The biodegradability of block copolymers is currently being studied.

There are several unanswered questions about the biosynthesis of block copolymers. First, the number of segments in the polymer chain is unknown. Although the in vitro analysis of PhaC_AR_ suggests that a diblock copolymer was synthesized, no direct evidence has been obtained. Additionally, the reaction mechanism generating block sequence has not been fully understood. In particular, it is necessary to analyze the mechanism by which the synthesis of the first segment promotes the synthesis of the second segment.

## Data Availability

All the data supporting the findings of this study are available within the cited references.
